# Development of a system for the detection of the inflammatory response induced by airborne fine particulate matter in rat tracheal epithelial cells

**DOI:** 10.1016/j.toxrep.2020.07.002

**Published:** 2020-07-17

**Authors:** Nobuyuki Yamagishi, Tomoki Yamaguchi, Takahisa Kuga, Masanari Taniguchi, Mohammad Shahriar Khan, Takahiro Matsumoto, Yuya Deguchi, Hiroaki Nagaoka, Keiji Wakabayashi, Tetsushi Watanabe

**Affiliations:** a Department of Analytics for Biomolecules, Faculty of Pharmaceutical Sciences, Setsunan University, 45-1 Nagaotoge-cho, Hirakata, Osaka 573-0101, Japan; b Department of Public Health, Kyoto Pharmaceutical University, 1 Misasagishichono-cho, Yamashina-ku, Kyoto 607-8412, Japan; c Faculty of Pharmaceutical Sciences, Nagasaki International University, 2825-7 Huis Ten Bosch, Sasebo, Nagasaki 859-3298, Japan; d Graduate Division of Nutritional and Environmental Sciences, University of Shizuoka, 52-1 Yada, Suruga-ku, Shizuoka 422-8526, Japan

**Keywords:** Airborne particulate matter, Endotoxin, Pro-inflammatory cytokines, ﬁne particle, Coarse particle

## Abstract

Exposure to airborne particulate matter (PM) is related to the increased risk of several diseases, including chronic and allergic rhinitis. We have previously shown that atmospheric endotoxin level was positively associated with the number of emergency department visits for asthma even after adjusting for meteorological factors, suggestive of the significant association between atmospheric endotoxin level and asthma exacerbation. Whether atmospheric endotoxin level is related to inflammatory response induction is, however, unclear. Here, we established stable cell lines to determine the promoter activity of the genes encoding pro-inflammatory cytokines such as tumor necrosis factor alpha, interleukin 6 (IL6), and IL33 by transfection of each reporter plasmid into rat tracheal epithelial EGV-4 T cells. These cells could measure the inflammatory response induced by endotoxin treatment more easily, rapidly, and sensitively than the conventional system using immunodetection assays. Furthermore, we revealed a relationship between atmospheric endotoxin level and inflammatory response induction. Thus, the system established herein may serve as a promising tool to monitor inflammatory response induced upon PM exposure.

## Highlights

• Atmospheric endotoxin level is related to inflammatory response induction.
• Stable cell lines established to determine the expression of pro-inflammatory genes.
• Our system serves monitoring of inflammatory response to airborne particulate matter.

## Graphical abstract

**Figure:**
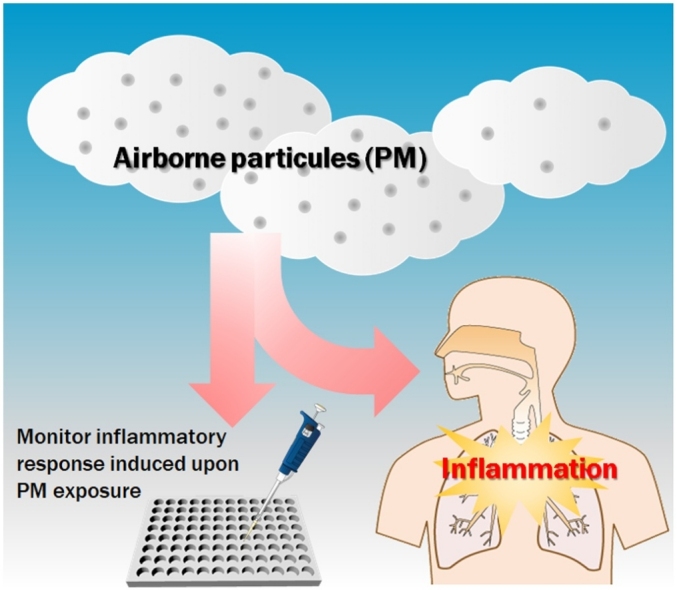

## 1 Introduction

Airborne particulate matter (PM) is one of the main components of polluted air and exposure to PM has been related to exacerbation of respiratory symptoms, increased risk of diabetes and cardiovascular diseases, and all-cause mortality [[1], [2], [3], [4], [5]]. Among PM, PM2.5 with an aerodynamic diameter equal to or smaller than 2.5 μm contributes the most to adverse health effects, owing to its ability to easily pass through the throat and nose and subsequently reach the alveolus [6]. Many epidemiological studies have shown that the exposure to PM is associated with the exacerbation of asthmatic symptoms and increase in the rate of asthma-related emergency department visits [[7], [8], [9], [10], [11], [12], [13]]. Considering these evidences, PM may result in toxicological effects through various mechanisms in the respiratory system, and prolongation of these biological events may lead to respiratory diseases such as chronic and allergic rhinitis and asthma.

PM comprises various materials such as organic chemicals (e.g., polycyclic aromatic hydrocarbons [PAHs]) and inorganic chemicals (e.g., sulfates, nitrates, and metals), which are involved in mediating toxicological effects [[14], [15], [16]]. In particular, PAHs and heavy metals are the main components that induce inflammatory response [17]. In addition, a variety of bioactive substances such as endotoxins are detected in PM [18]. Endotoxins, deﬁned as lipopolysaccharides (LPS), are major components of the outer membrane of gram-negative bacteria and well known to exert biological effects, including inflammation induction. Inhalation of endotoxins has been found to stimulate the alveolar macrophages and respiratory epithelial tissue to release cytokines or chemo-attractants to initiate an inflammatory cascade and exacerbate asthmatic symptoms [19,20]. The Institute of Medicine reviewed articles published from 2000 to 2013 on indoor environmental exposure and exacerbation of asthma, and found sufficient evidence on the association between indoor exposure to endotoxins and exacerbation of asthma [21]. However, the association between endotoxins in outdoor air and asthma exacerbation is incompletely investigated. We have recently demonstrated that atmospheric endotoxin level was positively associated with the number of emergency department visits for asthma even after adjustments for meteorological factors, suggestive of the significant association between atmospheric endotoxin level and asthma exacerbation [22]. Whether atmospheric endotoxin level is related to inflammatory response induction is, however, unclear.

Airway epithelial cells play a key role in inflammatory diseases of respiratory organs through the production and release of numerous pro-inflammatory cytokines, eventually leading to inflammatory response. To determine the relationship between atmospheric endotoxin level and inflammatory response following exposure to PM, we established reporter cell lines and estimated the expression of the genes encoding pro-inflammatory cytokines such as tumor necrosis factor alpha (TNFα), interleukin 6 (IL6), and IL33 following transfection of reporter plasmids into rat tracheal epithelial EGV-4 T cells. Using these cell lines, we show that the atmospheric endotoxin level in PM is related to the induction of inflammatory cytokine gene expression. These cells may serve as a convenient device to estimate the inflammatory potential of PM.

## 2 Experimental procedures

### 2.1 Collection of airborne particles and preparation of sample solution

Fine (aerodynamic diameter  ≤  2.5 μm, PM2.5) and coarse particles (aerodynamic diameter  ≥  2.5 μm) were collected on glass filters (Advantec Co., Ltd., Tokyo, Japan) and quartz filters (Pall Life Sciences, Port Washington, NY, USA), respectively, in the city of Sasebo (129.79 °E, 33.10 °N) using a high volume air sampler (HV1000R, Shibata Scientific Technology, Soka, Japan) equipped with an impactor (Shibata Scientific Technology) at a flow rate of 1 m^3^/min for 1 week per filter. The quartz and glass filters were preheated to 250 °C for 2 h before sampling for the estimation of endotoxin level. Following sample collection, the filters were stored at −80 °C until the preparation of sample solutions.

To quantify endotoxins in fine and coarse particles, 15 % of sample filter (corresponding to 1512 m^3^ of air) was extracted with endotoxin-free water containing 0.025 % Tween-20 using an ultrasonic apparatus for 30 min, as previously described [22,23]. The extract was centrifuged, and a portion of the supernatant was used for endotoxin analyses. A sample solution for luciferase reporter assay was extracted with distilled water from 15 % of the sample filter through ultra-sonication for 30 min, followed by centrifugation. The supernatant was lyophilized to obtain powder and resolved with culture medium before use in the luciferase reporter assay.

### 2.2 Quantitative analysis of endotoxin level in airborne particles

Atmospheric endotoxin level was analyzed by the kinetic chromogenic Limulus amebocyte lysate (LAL) method (Limulus Color KY Test Wako kit; Wako Pure Chemical Industries, Ltd., Osaka, Japan) according to the manufacturer’s instructions. All samples exceeded the detection limit (0.0005 EU/mL). The extract from a blank filter prepared by the method described above was below the detection limit. The recovery rates for spiked samples ranged between 50 % and 200 % that were deemed acceptable by the LAL assay kit.

### 2.3 Construction of reporter plasmids　

The reporter plasmids carrying the firefly luciferase cDNA driven by a human *TNFα*, *IL6*, and *IL33* gene promoters were constructed as follows. The 5′-flanking region of human *TNFα*, *IL6*, and *IL33* genes were the amplified forms of genomic DNAs derived from human HEK293 cells with polymerase chain reaction (PCR) using PrimeSTAR GXL DNA polymerase (TaKaRa BIO, Shiga, Japan) and specific primers as described in Table 1. The amplified DNA fragments were digested with *Kpn*I and *Xho*I and ligated into the complementary sites of pGL4.21[luc2P/puro] vector (Promega, Madison, WI, USA).

**Table 1:** Primer sequences for the reporter plasmids of cytokine genes.

Gene	Position	Orientation	Sequence
*TNFα*	nt −1335 to +79	Sense	5′-CGCGGTACCGCTGTCTGCTTGTGTGTGTG-3′
		Antisense	5′-CGCCTCGAGTGTCCTTTCCAGGGGAGAGAGA-3′
*IL6*	nt −800 to +105	Sense	5′-CGCGGTACCCAGTGAAACAGTGGTGAAGA-3′
		Antisense	5′-CGCCTCGAGTGGAGGGGAGATAGAGCTTC-3′
*IL33* V1	nt −2524 to +37	Sense	5′-CGCGGTACCCCATGCTTTCATCTTCATTC-3′
		Antisense	5′-CGCCTCGAGAGAGCTGCAGCTCTGTGTTC-3′
*IL33* V5	nt −1956 to +48	Sense	5′-CGCGGTACCTAAACTTCTGGGCTCAGGTG-3′
		Antisense	5′-CGCCTCGAGGCTGGTCTCAGATGATGAGG-3′

### 2.4 Cell culture and transfection

Rat tracheal epithelial EGV-4 T cells (JCRB0229) were obtained from the Japanese Cancer Research Resource Bank and maintained at 37 °C and 5% CO_2_ in Ham's F12 medium supplemented with 10 % fetal bovine serum.

To establish stable reporter cell lines, the reporter plasmids for *TNFα*, *IL-6*, and *IL-33* genes were transfected into EGV-4 T cells using Lipofectamine 2000 reagent (Invitrogen, Carlsbad, CA, USA) according to the manufacturer’s instructions. After 48 h from transfection, the cells were maintained in a growth medium containing 0.5 μg/mL puromyxin for 3 weeks for the selection of puromycin-resistant cells. The surviving cell clones were isolated and stable cell lines with a reporter plasmid for either human *TNFα*, *IL6*, or *IL33* gene promoter were established.

### 2.5 Measurement of promoter activity of cytokine genes

EGV-4 T cells transfected with reporter plasmids for pro-inflammatory cytokines (5 × 10^4^ cells/100 μL) were seeded in each well of a 96-well plate and treated with LPS (control standard endotoxin from *Escherichia coli* UKT-B, WAKO Pure Chemicals, Osaka, Japan) or airborne particles for 2−12 h at 37 °C. In the experiments using polymycin B (PMB), an endotoxin neutralizer, airborne particles corresponding to 80 m^3^ of air were treated with PMB (final concentration at 50 μg/mL) in 1 mL of culture medium for 1 h at 37 °C before exposure to cells. The cells were washed thrice with phosphate-buffered saline (PBS) and lysed in 30 μL of Glo Lysis buffer (Promega). The cell lysates were centrifuged at 20,000 ×*g* for 5 min, and the supernatants were recovered as cell extracts. Aliquots (2 μL) of the extracts were added to 25 μL of luciferase assay reagent (Promega), and the luciferase activity was measured using a luminometer (model TD-20/20, Turner Designs, Sunnyvale, CA, USA). The luciferase activity of each sample was normalized to protein concentration and expressed relative to the control.

### 2.6 Western blot analysis

EGV-4 T cells were seeded into each well of 24-well plates at a density of 4 × 10^5^ cells/mL. After 24 h of incubation, the cells were treated with different concentrations of LPS for 24 h. The cell culture media (500 μL) were recovered and centrifuged at 2000 ×*g* for 10 min. The supernatant was lyophilized to obtain powder, resolved with 50 μL of 4× sodium dodecyl sulfate (SDS) sample buffer (250 mM Tris−HCl [pH 6.8], 40 % glycerol, 8% SDS, 20 % 2-mercaptoethanol, and 0.005 % bromophenol blue), and boiled for 5 min. The cells were lysed with 200 μL of SDS sample buffer (62.5 mM Tris−HCl [pH 6.8], 10 % glycerol, 2% SDS, 5% 2-mercaptoethanol, and 0.00125 % bromophenol blue) and boiled for 5 min. Aliquots of cell culture supernatant and cell extract were subjected to SDS polyacrylamide gel electrophoresis, and the separated protein bands were transferred onto Immobilon-P transfer membranes (Merck Millipore, Tullagreen, Carrigtwohill, Co. Cork, Ireland). Immunodetection was performed with a chemiluminescent detection method (Nacalai Tesque, Kyoto, Japan). Antibodies used in this study were as follows: anti-TNFα monoclonal antibody (52B83, Santa Cruz Biotechnology), anti-IL6 monoclonal antibody (10E5, Santa Cruz Biotechnology), anti-IL33 monoclonal antibody (EPR17831, Abcam, Cambridge, UK), and anti-glyceraldehyde 3-phosphate dehydrogenase (GAPDH) monoclonal antibody (5A12, Wako Pure Chemical Industries, Osaka, Japan).

### 2.7 Cell viability assay

EGV-4 T cells (1 × 10^5^ cells/well) seeded in 48-well plates were treated with PM collected in Sasebo, Japan, for 24 h at 37 °C. Cell viability was examined with the 3-(4,5-dimethylthiazol-2-yl)-2,5-diphenyltetrazolium bromide (MTS) assay using CellTiter 96 AQueous one solution cell proliferation assay (Promega) in accordance with the manufacturer’s instructions.

### 2.8 Statistical analysis

Statistical analysis were performed using BellCurve for Excel (Social Survey Research Information Co., Ltd, Tokyo, Japan). All values are expressed as the mean ± standard deviation (SD). Data were statistically evaluated using a one-way or two-way analysis of variance (ANOVA) followed by Tukey’s test. A value of *p* <  0.05 was considered statistically significant.

## 3 Results and discussion

### 3.1 Establishment of stable cell lines to determine the promoter activity of pro-inflammatory cytokine genes

**Fig. 1:**
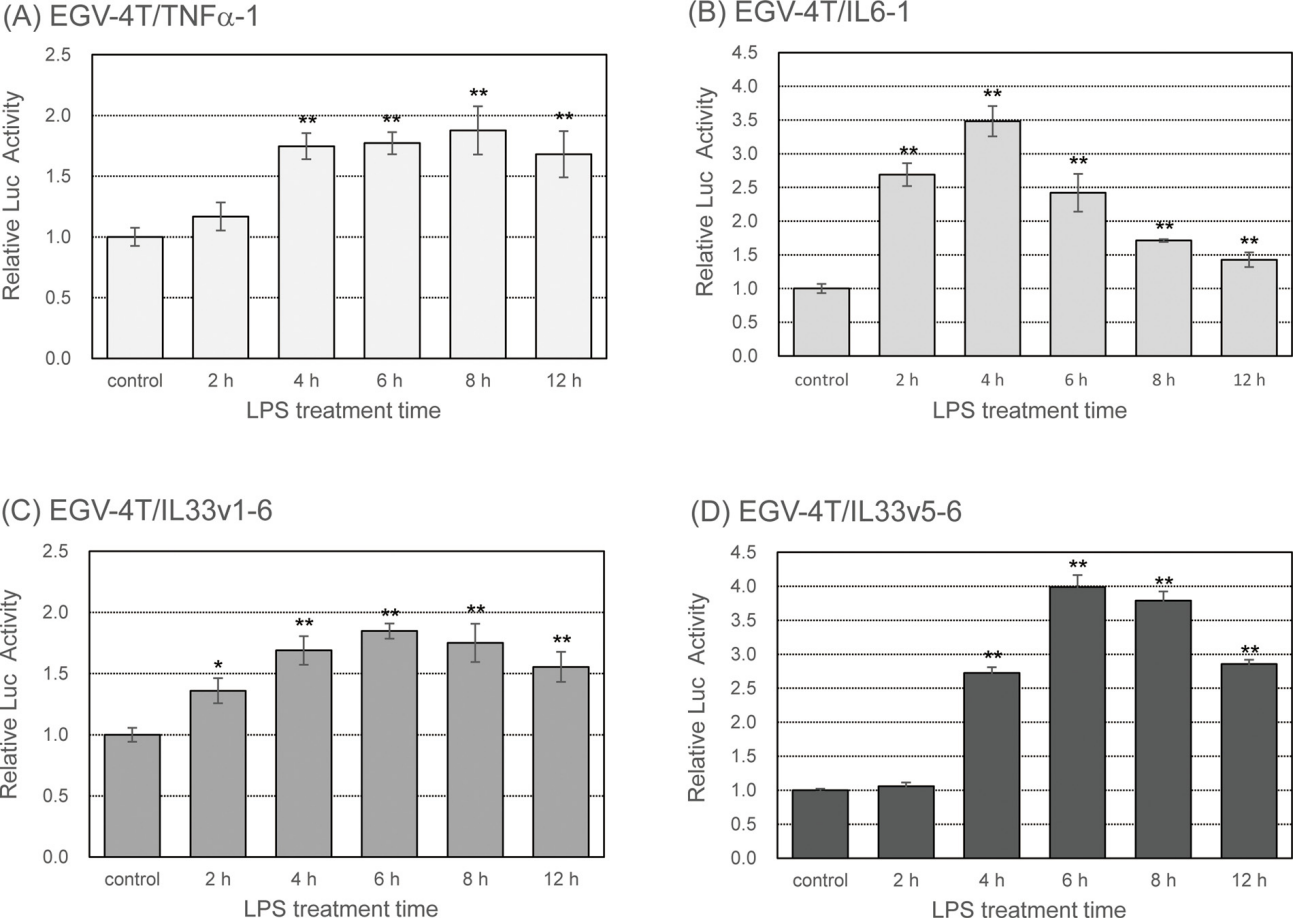
Rat tracheal epithelial EGV-4 T cells transfected with a reporter plasmid to determine the promoter activity of pro-inflammatory cytokine genes (EGV-4 T/TNFα-1, EGV-4 T/IL6-1, EGV-4 T/IL33v1-6, and EGV-4 T/IL33v5-6 cells) were stimulated with 10 EU/mL LPS for various time points. Luciferase activity was measured to determine the promoter activity of *TNFα*, *IL6*, and *IL33* genes. Relative activity is shown as a ratio to luciferase activity of untreated control cells. Each value represents the mean ± SD (n = 5). *p < 0.05, **p < 0.01 versus untreated control.

Pro-inflammatory cytokines such as TNFα and IL6 are known to be involved in the initiation and regression of immune responses [24,25]. To investigate the PM-induced inflammatory response in tracheal epithelial cells, we established stable cell lines and determined the promoter activity of some pro-inflammatory cytokine genes such as TNFα, IL6, and IL33. We transfected each reporter plasmid into rat tracheal epithelial EGV-4 T cells and characterized these cells by stimulating them with 10 EU/mL LPS for various time points, followed by the evaluation of the promoter activity of pro-inflammatory cytokine genes. As shown in Fig. 1, the promoter activity of *TNFα*, *IL6*, and *IL33* genes increased after treatment with LPS, reached maximum values at 4−8 h after the treatment with LPS, and decreased thereafter.

**Fig. 2:**
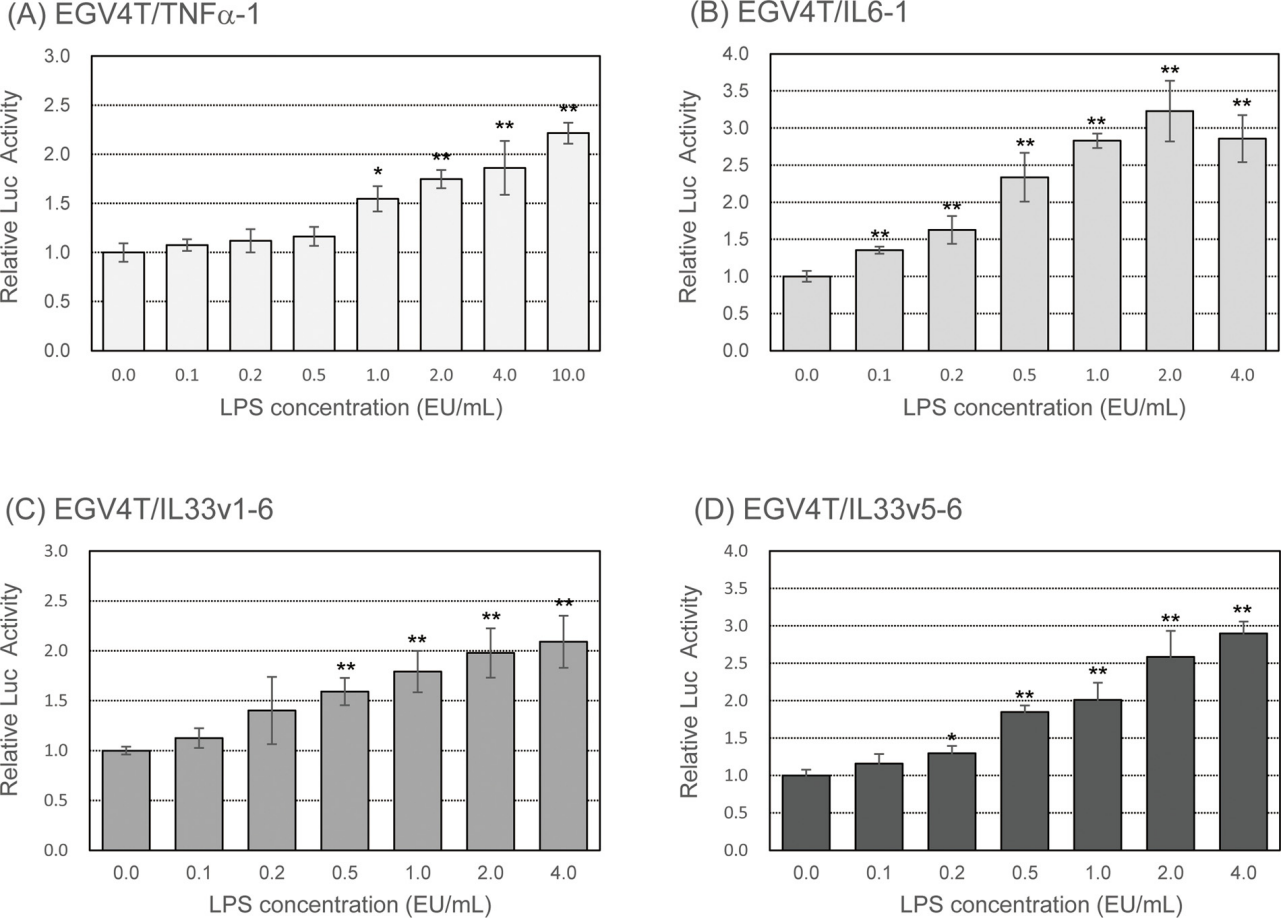
EGV-4 T/TNFα-1, EGV-4 T/IL6-1, EGV-4 T/IL33v1-6, and EGV-4 T/IL33v5-6 cells were stimulated with LPS at indicated concentrations for 6 h. Luciferase activity was measured to determine the promoter activity of pro-inflammatory cytokine genes. Relative activity is shown as a ratio to luciferase activity of untreated control cells. Each value represents the mean ± SD (n = 6). *p < 0.05, **p < 0.01 versus untreated control.

**Fig. 3:**
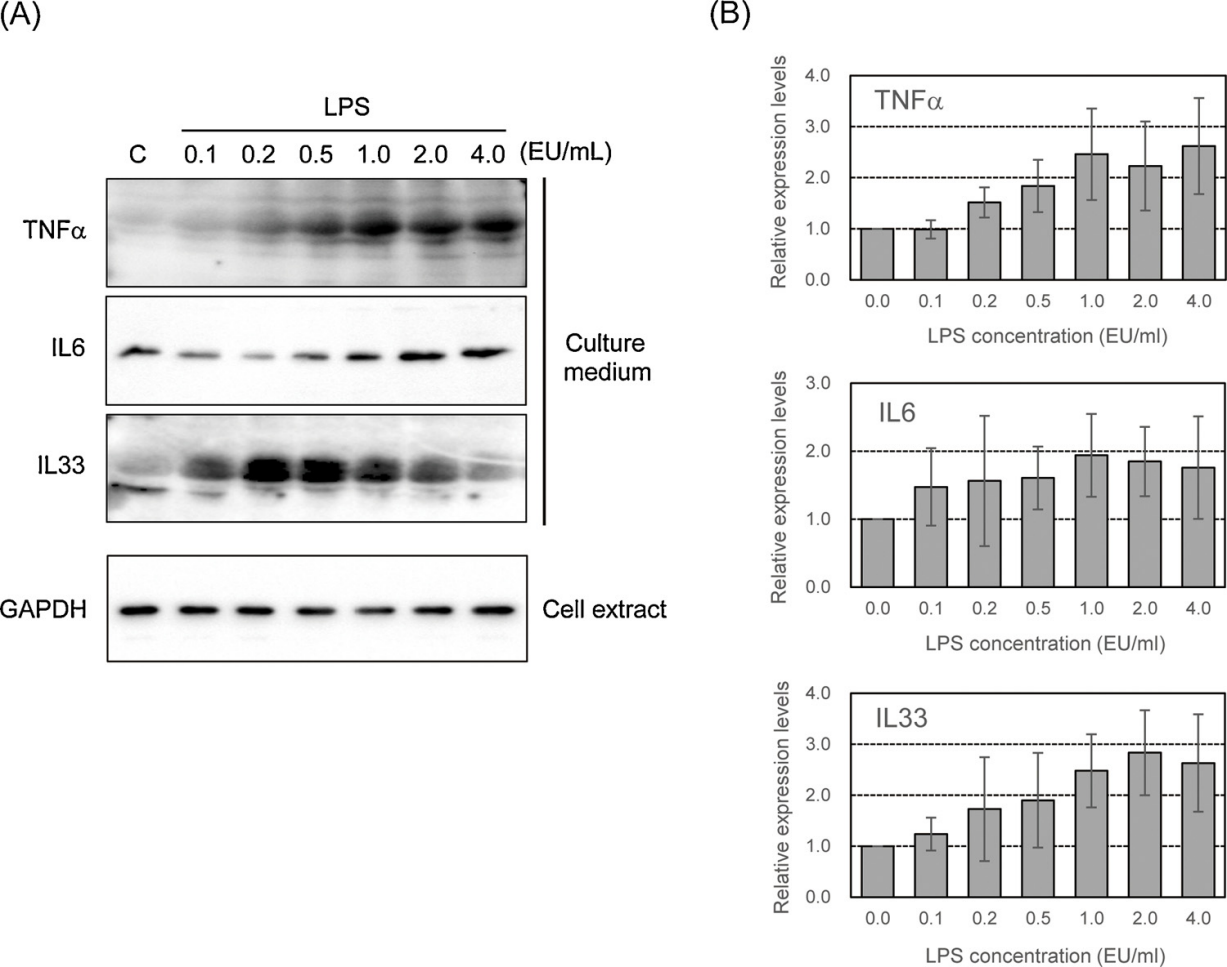
A) Detection of LPS-induced secretion of pro-inflammatory cytokines in EGV-4 T cells. Cells were stimulated with LPS at indicated concentrations for 24 h. The protein levels of TNFα, IL6, and IL33 in cell culture supernatants and cell extracts were analyzed with western blotting. (B) The density of bands was quantified by densitometry, and was corrected with the density of GAPDH as loading control. Relative levels of TNFα, IL6, and IL33 are shown as a ratio to respected levels of untreated cells. Each value represents the mean ± SD (n = 7).

We examined the induction of promoter activity of pro-inflammatory cytokine genes following treatment with LPS at various concentrations for 6 h. As shown in Fig. 2, the promoter activity of *TNFα*, *IL6*, and *IL33* genes increased upon treatment with LPS in a dose-dependent manner. We also measured the production of pro-inflammatory cytokines in LPS-stimulated parental EGV-4 T cells with western blotting. As shown in Fig. 3, the secretion of TNFα and IL6 increased upon LPS treatment in a dose-dependent manner from 0.1–4.0 EU/mL. IL33 secretion also increased after treatment with LPS in a dose-dependent manner at concentrations from 0.1 to 0.5 EU/mL and gradually decreased.

Thus, the stable cell lines that estimated the promoter activity of pro-inflammatory cytokine genes could measure the inflammatory response more easily, rapidly, and sensitively than the conventional system using an immunodetection assay and may be promising for the monitoring of PM-induced inflammatory response.

### 3.2 Increase in inflammatory response in tracheal epithelial EGV-4 T cells following exposure to PM collected in Sasebo, Japan

**Fig. 4:**
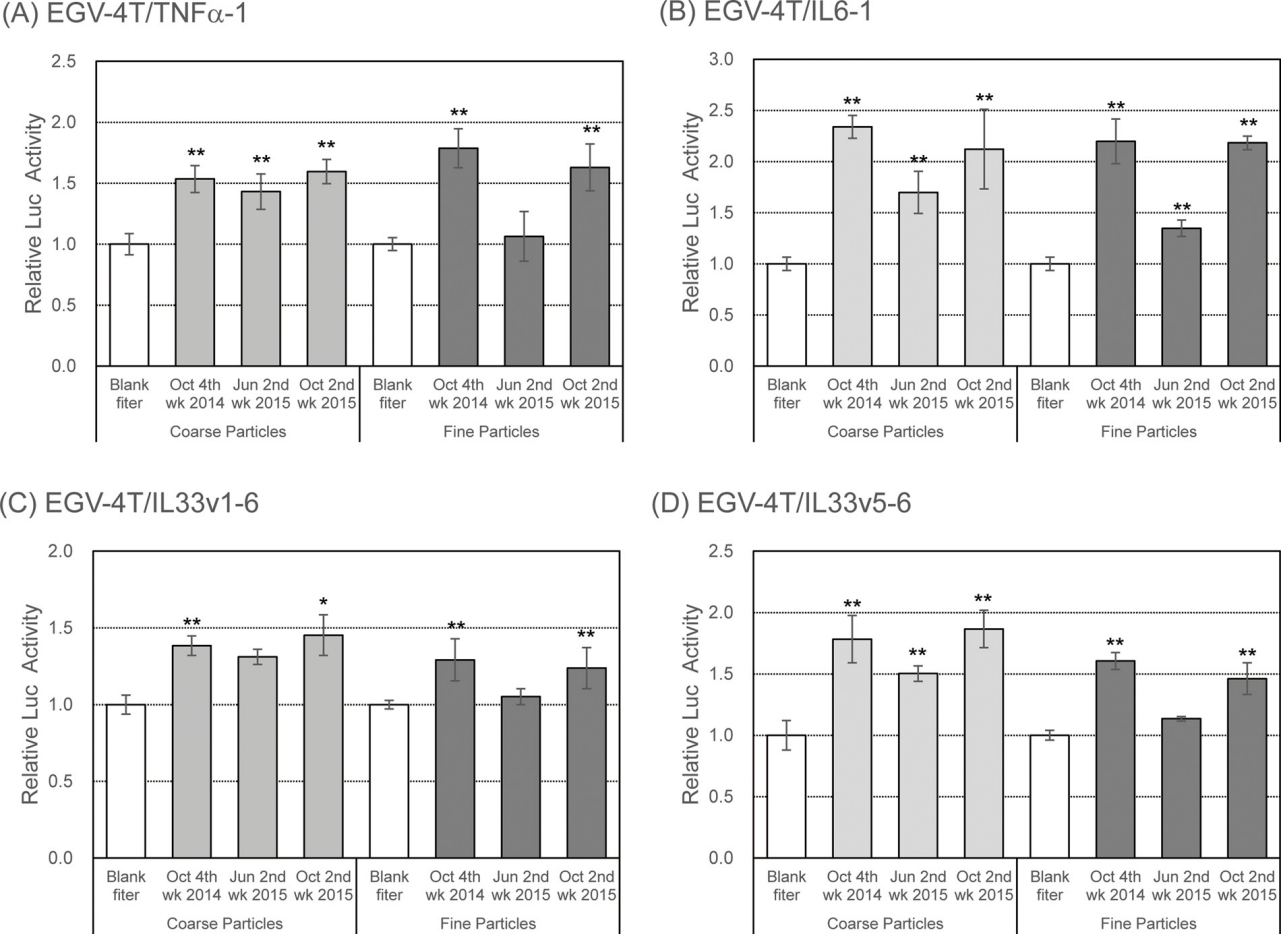
Increase in inflammatory response of EGV-4 T cells following exposure to PM collected in Sasebo, Japan. EGV-4 T/TNFα-1, EGV-4 T/IL6-1, EGV-4 T/IL33v1-6, and EGV-4 T/IL33v5-6 cells were exposed to coarse or fine particles corresponding to 80 m^3^ of air in 1 mL culture medium. After 6 h, the luciferase activity was measured to determine the promoter activity of pro-inflammatory cytokine genes. Relative activity is shown as a ratio to luciferase activity of cells treated with the extract from the blank filter. Each value represents the mean ± SD (n = 6). *p < 0.05, **p < 0.01 versus the extract from the blank filter.

We have previous shown that inhaled PM may play a key role in inflammatory diseases through the production and release of numerous inflammatory cytokines, eventually leading to inflammatory diseases such as chronic and allergic rhinitis [[26], [27], [28], [29]]. To examine the impact of PM on inflammatory cytokine production, the cells transfected with reporter plasmids for pro-inflammatory cytokines were exposed to aqueous extracts of PM. As shown in Fig. 4, the promoter activity for *TNFα*, *IL6*, and *IL33* increased upon exposure to both coarse particles and ﬁne particles in October. Interestingly, the induction of promoter activity of pro-inflammatory cytokine genes was detected following exposure to coarse particles, but not ﬁne particles, in June.

Table 2 shows the concentrations of particles and endotoxins in coarse particles and fine particles at the same time period. No significant difference was observed in the concentrations of coarse particles and fine particles at any month. On the other hand, the concentration of endotoxins in coarse particles was higher than that in fine particles. These results are consistent with those previously reported [22,[30], [31], [32]]. In addition, the concentration of endotoxins in October was approximately 2–5 times higher than that detected in June. Atmospheric endotoxin level in PM seemed to be positively correlated with the induction of pro-inflammatory cytokine gene expression.

**Table 2:** Concentrations of endotoxins in coarse and fine particles collected in Sasebo, Japan.

Sample	Coarse particles		Fine particles	
	Particle (μg/m^3^)	Endotoxin (EU/m^3^)	Particle (μg/m^3^)	Endotoxin (EU/m^3^)
October 4^th^ week, 2014	13.3	0.0412	14.6	0.0160
June 2^nd^ week, 2015	16.0	0.0244	13.2	0.0038
October 2^nd^ week, 2015	18.0	0.0447	17.5	0.0156

**Fig. 5:**
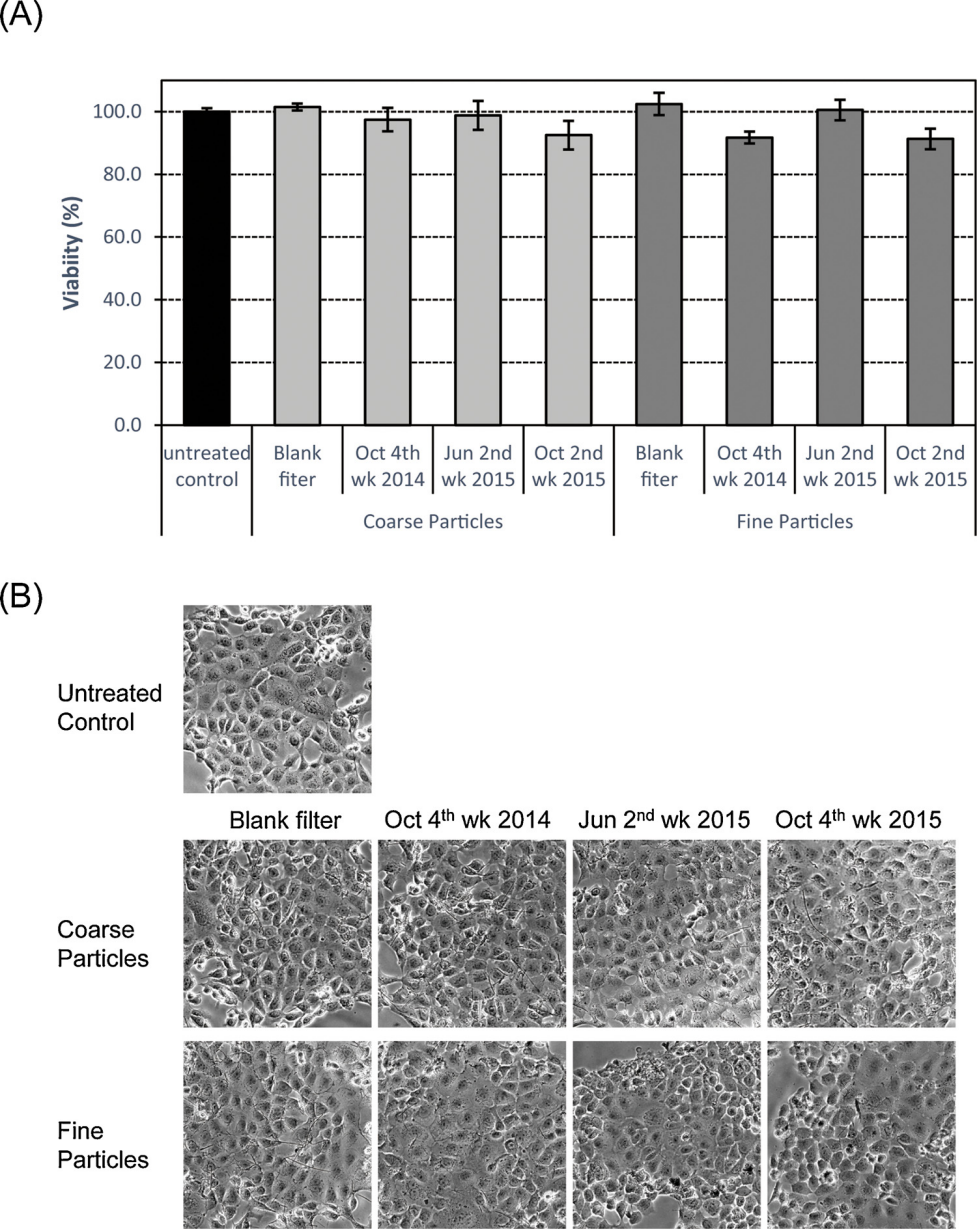
Viability of EGV-4 T cells exposed to PM collected in Sasebo, Japan. Cells were exposed to coarse or fine particles corresponding to 80 m^3^ of air in 1 mL of culture medium. (A) After 24 h, cell viability was measured with MTS assay. Each value represents the mean ± SD (n = 4). (B) Cell morphology following exposure to coarse or fine particles for 24 h was observed by phase-contrast microscopy.

To explore the cytotoxicity of PM, we examined the effects of PM on the viability and morphology of EGV-4 T cells with MTS assay. As shown in Fig. 5A, MTS activity was unaffected after treatment of cells with extracts of coarse particles or ﬁne particles for 24 h at different concentrations. Furthermore, we failed to notice any significant changes in cellular morphology upon exposure to PM for 24 h (Fig. 5B). Therefore, PM may not cause any drastic cytotoxicity at concentrations used in our study.

### 3.3 Important role of endotoxins in the inflammatory response induced upon exposure to PM collected in Sasebo, Japan

**Fig. 6:**
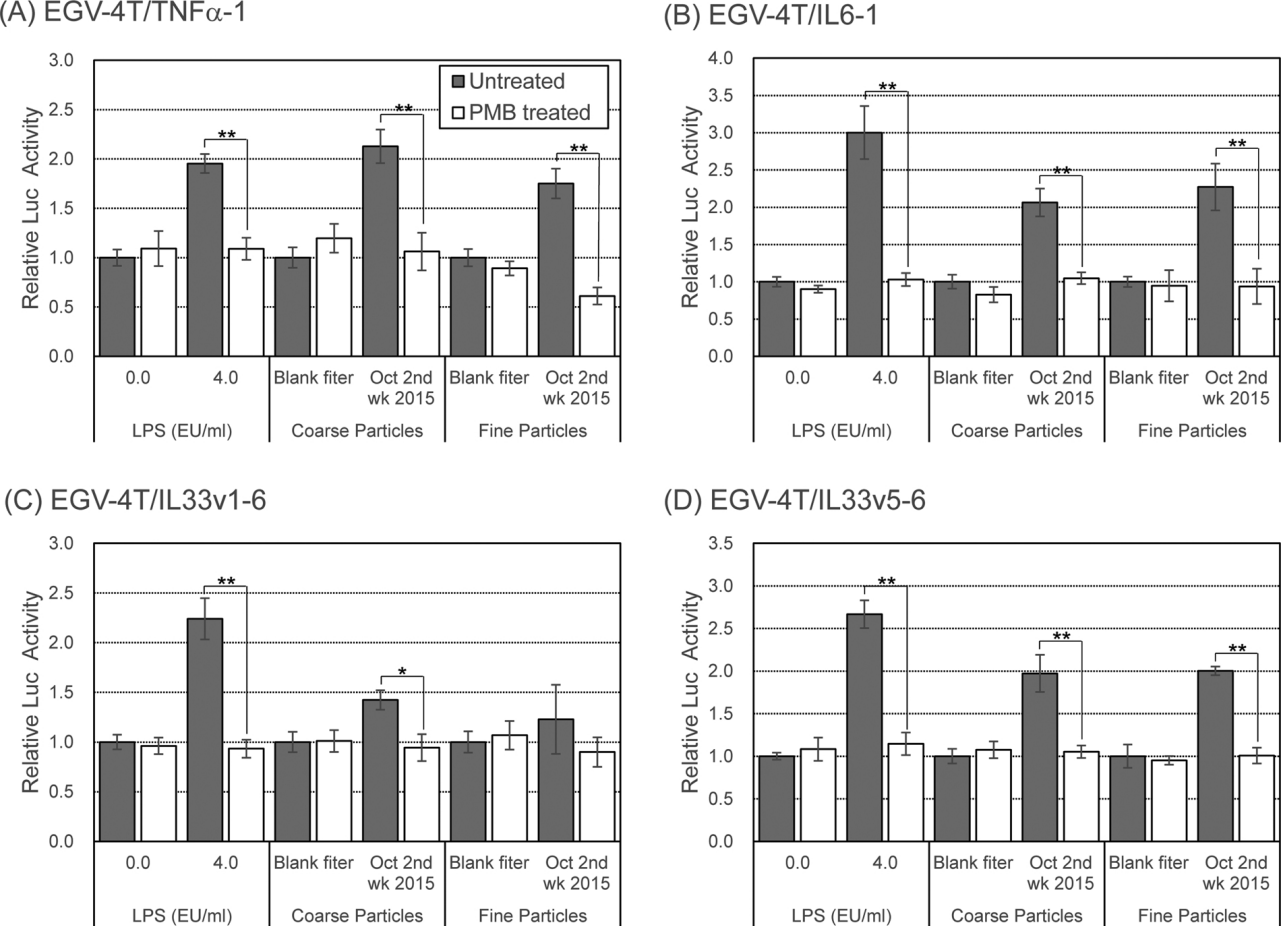
Effects of PMB treatment on the induction of the promoter activity of pro-inflammatory cytokine genes in EGV-4 T cells upon exposure to PM collected in Sasebo, Japan. Coarse or fine particles corresponding to 80 m^3^ of air were pre-treated with PMB (50 μg/mL) in 1 mL of culture medium for 1 h at 37 °C. EGV-4 T/TNFα-1, EGV-4 T/IL6-1, EGV-4 T/IL33v1-6, and EGV-4 T/IL33v5-6 cells were exposed to PM or PMB-treated PM for 6 h, and the luciferase activity was measured to determine the promoter activity of pro-inflammatory cytokine genes. Relative activity is shown as a ratio to luciferase activity of cells treated with the extract from the blank filter. Each value represents the mean ± SD (n = 6). *p < 0.05, **p < 0.01 versus each control.

We have previously shown that atmospheric endotoxin level was positively associated with the number of emergency department visits for asthma even after adjustment for meteorological factors, suggesting that atmospheric endotoxin level was significantly associated with asthma exacerbation [22]. To examine the contribution of endotoxin to the induction of inflammatory response following PM exposure, the cells carrying the reporter plasmids for pro-inflammatory cytokines were exposed to PM treated with polymyxin B (PMB, an endotoxin neutralizer). As expected, PMB treatment completely suppressed the increase in the promoter activity of pro-inflammatory cytokine genes following treatment with LPS or PM, indicative of the substantial involvement of endotoxins in the inflammatory reaction following PM exposure (Fig. 6).

Thus, our results highlight the important role of endotoxins in mediating inflammatory response following exposure to PM collected in Sasebo. However, the promoter activity of pro-inflammatory cytokine genes was induced after the treatment of cells with same levels of ﬁne and coarse particles. The endotoxin level was lower in ﬁne particles than in coarse particles. PM contains hundreds of different types of organic and inorganic chemical elements, which are involved in mediating toxicological effects [[14], [15], [16]], and the levels of these chemicals may correlate with the inflammatory response after exposure to PM. For instance, many studies have shown that metal components in PM are associated with pulmonary toxicity [1,33,34]. There is a possibility of synergistic effects existing between these organic and metal components. Future studies are warranted to assess the correlation between the chemical composition of PM and adverse health effects.

## 4 Conclusions

We demonstrated that the atmospheric endotoxin level was related to the inflammatory response induction. The cells established in this study may be used to estimate the inflammatory response in an easy, rapid, and highly sensitive manner as compared with the conventional systems using immunodetection assays. Although specific components in charge of PM-induced inflammatory response have not been fully investigated, our analytical system provides an insight into the monitoring of inflammatory response induced by PM exposure.

## CRediT authorship contribution statement

**Nobuyuki Yamagishi:** Conceptualization, Data curation, Formal analysis, Funding acquisition, Investigation, Methodology, Project administration, Supervision, Validation, Visualization, Writing - original draft, Writing - review & editing. **Tomoki Yamaguchi:** Investigation, Validation, Visualization, Writing - original draft. **Takahisa Kuga:** Project administration, Validation, Writing - review & editing. **Masanari Taniguchi:** Formal analysis, Project administration, Validation, Writing - review & editing. **Mohammad Shahriar Khan:** Resources. **Takahiro Matsumoto:** Resources, Writing - review & editing. **Yuya Deguchi:** Resources, Writing - review & editing. **Hiroaki Nagaoka:** Project administration, Resources, Writing - review & editing. **Keiji Wakabayashi:** Project administration, Writing - review & editing. **Tetsushi Watanabe:** Conceptualization, Data curation, Funding acquisition, Project administration, Resources, Writing – review & editing.

## Declaration of Competing Interest

The authors declare that they have no known competing financial interests or personal relationships that could have appeared to influence the work reported in this paper.
